# Health resource utilization and cost before versus after initiation of second-generation long-acting injectable antipsychotics among adults with schizophrenia in Alberta, Canada: a retrospective, observational single-arm study

**DOI:** 10.1186/s12888-022-04075-y

**Published:** 2022-07-02

**Authors:** Kai On Wong, Scott W. Klarenbach, Karen J. B. Martins, Pierre Chue, Serdar M. Dursun, Mark Snaterse, Alexis Guigue, Helen So, Huong Luu, Khanh Vu, Lawrence Richer

**Affiliations:** 1 University of Alberta, Edmonton, AB Canada; 2 Faculty of Medicine and Dentistry, University of Alberta, Edmonton, AB Canada; 3 Department of Medicine, University of Alberta, 11-12R Clinical Sciences Building, 11350-83rd avenue, Edmonton, AB T6G 2B7 Canada; 4 Department of Psychiatry, University of Alberta, Edmonton, AB Canada; 5 Alberta Health Services, Edmonton, AB Canada; 6 University of Calgary, Calgary, AB Canada; 7 Department of Pediatrics, University of Alberta, Edmonton, AB Canada

**Keywords:** Antipsychotics, Long-acting injection, Schizophrenia, Healthcare resource utilization, Healthcare cost, Community treatment order, Retrospective, Administrative data

## Abstract

**Background:**

Long-acting injectable (LAI) antipsychotics, along with community treatment orders (CTOs), are used to improve treatment effectiveness through adherence among individuals with schizophrenia. Understanding real-world medication adherence, and healthcare resource utilization (HRU) and costs in individuals with schizophrenia overall and by CTO status before and after second generation antipsychotic (SGA)-LAI initiation may guide strategies to optimize treatment among those with schizophrenia.

**Methods:**

This retrospective observational single-arm study utilized administrative health data from Alberta, Canada. Adults (≥ 18 years) with schizophrenia who initiated a SGA-LAI (no use in the previous 2-years) between April 1, 2014 and March 31, 2016, and had ≥ 1 additional dispensation of a SGA-LAI were included; index date was the date of SGA-LAI initiation. Medication possession ratio (MPR) was determined, and paired t-tests were used to examine mean differences in all-cause and mental health-related HRU and costs (Canadian dollars), comprised of hospitalizations, physician visits, emergency department visits, and total visits, over the 2-year post-index and 2-year pre-index periods. Analyses were stratified by presence or absence of an active CTO during the pre-index and/or post-index periods.

**Results:**

Among 1,211 adults with schizophrenia who initiated SGA-LAIs, 64% were males with a mean age of 38 (standard deviation [SD] 14) years. The mean overall antipsychotic MPR was 0.39 (95% confidence interval [CI] 0.36, 0.41) greater during the 2-year post-index period (0.84 [SD 0.26]) compared with the 2-year pre-index period (0.45 [SD 0.40]). All-cause and mental health-related HRU and costs were lower post-index versus pre-index (*p* < 0.001) for hospitalizations, physician visits, emergency department visits, and total visits; mean total all-cause HRU costs were $33,788 (95% CI -$38,993, -$28,583) lower post- versus pre-index ($40,343 [SD $68,887] versus $74,131 [SD $75,941]), and total mental health-related HRU costs were $34,198 (95%CI -$39,098, -$29,297) lower post- versus pre-index ($34,205 [SD $63,428] versus $68,403 [SD $72,088]) per-patient. Forty-three percent had ≥ 1 active CTO during the study period; HRU and costs varied according to CTO status.

**Conclusions:**

SGA-LAIs are associated with greater medication adherence, and lower HRU and costs however the latter vary according to CTO status.

**Supplementary Information:**

The online version contains supplementary material available at 10.1186/s12888-022-04075-y.

## Background

Schizophrenia is a serious chronic mental illness affecting approximately 0.3% to 1.0% of the global population [1, 2]. Although prevalence is relatively low, the burden of disease is substantial [2]. In Europe, the annual direct healthcare cost per patient has been reported to range from €533 to €13,704, with inpatient hospitalizations representing the largest proportion of costs in the majority of countries [3]. The total annual economic burden attributed to schizophrenia in Canada was estimated at $6.85 billion CDN in 2004 [1].

Pharmacotherapy with antipsychotics is considered to be the cornerstone of symptom treatment and relapse prevention among individuals with schizophrenia [4–6]. However, nonadherence to antipsychotics is common and associated with worsening functional outcomes, higher admission to the hospital and emergency department (ED), and higher rates of relapse and suicide [7–9]. Conversely, adherence to antipsychotics has been found to be associated with better long-term clinical outcomes [7]. Long-acting injectable (LAI) formulations of first- and second-generation antipsychotics (FGA and SGA, respectively) were developed with the goal of improving medication adherence [10, 11]. Clinical guidelines recommend LAIs over oral formulations of antipsychotics for individuals who display nonadherence, have frequent relapses, or pose a safety risk to others [6, 12, 13]. Although guidelines also recommend LAI antipsychotics be offered as an option in line with personal preference, it is noted that certain circumstances may not allow for this option, such as in the case of community treatment orders (CTOs) [6]. CTOs are available for use in over 75 jurisdictions worldwide; admissibility criteria and provisions of care can vary. In Alberta, a CTO is intended to assist with treatment adherence, including medications and attending health care appointments and other community support services, in those with mental illness who would otherwise decompensate, likely cause harm to themselves or others, or suffer substantial deterioration or impairment in the absence of continued treatment.

While CTOs are a common component of clinical practice in the treatment of schizophrenia, few studies investigating the effects of LAI antipsychotics on healthcare resource utilization (HRU) and associated costs have incorporated CTO information [14]. Additionally, it has been suggested that nonadherence and subsequent associated consequences may take time to develop, and that a 2-year observation period may result in a markedly higher likelihood of detecting a difference compared with a 1-year period [15]. Therefore, the aim of this observational study was to examine and compare real-world antipsychotic medication adherence, and HRU and associated costs between the 2-year period before and after SGA-LAI initiation among adults with schizophrenia; subgroup analysis was performed based on CTO status.

## Methods

This retrospective, non-interventional, single arm study is reported according to the STROBE guidelines [16]. Ethics approval (Pro00086993) was obtained from the institutional review board at the University of Alberta, Canada. This is a study of administrative data without any intervention. No study participants were placed at risk as a result of this study, and informed consent was not required.

### Data source

Administrative data from the Discharge Abstract Database (DAD), National Ambulatory Care Reporting System (NACRS), Practitioner Claims, and Pharmaceutical Information Network (PIN) were used. CTO data was obtained from Alberta Health Services. Data were linked to the Population Registry, which contains demographic information for all Albertans with Alberta Health Care Insurance Plan (AHCIP) coverage; all Alberta residents are eligible for AHCIP and over 99% participate [17]. The DAD includes demographic, administrative, diagnostic, procedural, and resource intensity weighting (RIW) information on all patients discharged from hospital; diagnostic data contains a most responsible diagnosis and up to 24 secondary diagnostic codes using the International Classification of Disease—Version 10—Canadian Enhancement (ICD-10-CA) codes. NACRS includes patient and RIW information in facility-based ambulatory care centers, including the ED; ICD-10-CA codes are used and contains a most responsible diagnosis field and up to 9 secondary diagnostic codes. Practitioner Claims includes patient, provider, service and direct billing information on fee-for-service, alternative payment plan physician billing and shadow billing; up to 3 ICD—Version 9—Clinical Modification (ICD-9-CM; Alberta specific) diagnostic codes can be used per visit. PIN contains information on dispensed prescription medications from community pharmacies.

### Cohort selection

The population of interest were adults with schizophrenia who initiated SGA-LAIs in Alberta within the inclusion period from April 1, 2014 to March 31, 2016. Eligibility criteria included those who: 1) initiated a SGA-LAI (≥ 1 dispensation within no use in the previous 2-years) during the inclusion period (index date was the first date of dispensation of either aripiprazole, paliperidone, or risperidone, which were the three approved SGA-LAIs in Alberta during this timeframe), 2) had ≥ 1 additional SGA-LAI dispensation during the 2-year post-index period, 3) met the case definition for schizophrenia within 9 years prior to the index date [18], 4) were ≥ 18-years on the index date 5) had AHCIP coverage over the study period (between April 1, 2014 and March 31, 2018). The following case algorithm was used to identify schizophrenia: ≥ 1 hospitalization or ≥ 3 physician visits with a diagnostic code for schizophrenia and/or related condition within a 3-year period [19]; the specific diagnostic codes used (see Additional file 1) were based on previous case validation studies and input experts [18, 19].

CTO status was defined by the presence or absence of ≥ 1 active CTO during the 2-year pre-index and/or post-index periods. The resultant four CTO categories included individuals: 1) without any active CTOs in both the pre- and post-index periods (pre = no / post = no), 2) with ≥ 1 active CTO in both the pre- and post-index periods (pre = yes / post = yes), 3) with ≥ 1 active CTO during the pre-index, but not the post-index period (pre = yes / post = no), and 4) with no active CTO during the pre-index period, but ≥ 1 active CTO during the post-index period (pre = no / post = yes).

### Study measures

Study measures were presented for the overall cohort and by CTO status. Baseline characteristics on the index date included age, sex, urban or rural residence (determined by the second digit of the postal code), and the SGA-LAI that was initiated. A Charlson Comorbidity Index (CCI) score was determined during the 2-year period before the index date; the score was based on the presence or absence of 17 individual comorbid conditions that were weighted according to their potential for influencing mortality [20].

Study outcomes included antipsychotic medication adherence, all-cause and mental health-related HRU, and associated healthcare costs that were measured and compared between the 2-year pre-index and post-index periods for all subjects within the overall cohort and the CTO subgroups. The number of individuals who received ≥ 1 dispensation for an antipsychotic medication was determined, and medication possession ratio (MPR) was calculated based on the number of antipsychotic medication days of supply during the 2-year pre- and post-index periods among all subjects (within the overall cohort and within the CTO subgroups), and presented overall; for completeness specific formulations (oral or injectable) and generations (first or second) of antipsychotic medications were also presented (see Additional file 2 for a complete listing of antipsychotic medications that were included). HRU included all-cause and mental health-related (ICD-9 290–319 from any diagnostic field; ICD-10 F01-F99 from the most responsible diagnostic field) hospitalizations, physician visits, ED visits, and total visits [21, 22]. Multiple physician visits per day per person were considered as one visit per day per person. The cost for each hospitalization and ED visit was determined by multiplying the RIW value of the visit with the 2016/2017 Canadian Institute for Health Information cost of a standard hospital stay (CSHS) for Alberta [23]. RIW is a measure to estimate HRU and represents the relative value of resources that a given patient, contingent on diagnostic case-mix, would be expected to consume relative to a standard patient; CSHS provides standardized average costs incurred through the direct care of a standard hospital or ambulatory care visit. Direct billing costs were used for physician visits. Costs are presented in Canadian dollars ($CDN) and reflect healthcare costs incurred over the study period.

### Statistical analyses

Descriptive statistics are reported as means and standard deviations (SD) or counts and percentages, where appropriate. Paired t-tests were used to test for statistically significant differences between the pre- and post-index periods for HRU and associated costs; costs were also compared using Wilcoxon signed-rank tests and medians and interquartile ranges (IQR) were reported. A conventional alpha of 0.05 and a two-tailed level of significance were used; accompanying 95% confidence intervals (CI) are reported. Statistical analyses were performed using Python version 3.6.5 and STATA version 17.0.

## Results

### Cohort selection

**Fig. 1:**
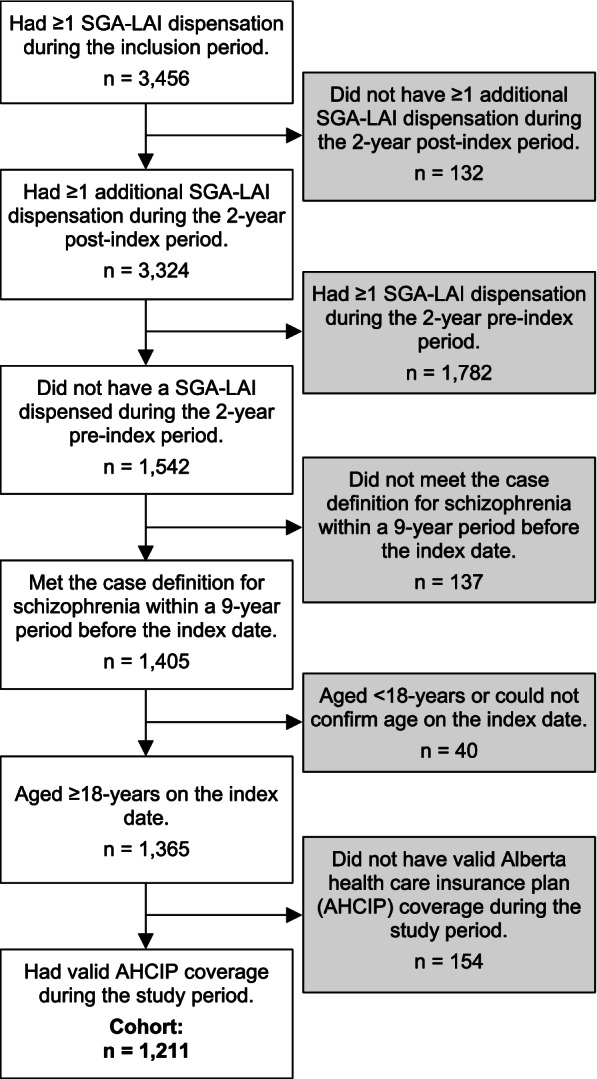
Flow diagram of cohort selection. Abbreviations: SGA-LAI = second-generation antipsychotic long-acting injectable

Of the 3,456 individuals who received a SGA-LAI dispensation during the inclusion period, 1,211 met eligibility criteria (Fig. 1). Within the cohort, 43% received at least one active CTO during the study period, among whom the majority (80%) persisted with the same CTO status throughout the pre- and post-index periods (pre = no / post = no [*n* = 689; 57%]; pre = yes / post = yes [*n* = 275; 23%]), and 20% had a CTO status change (pre = yes / post = no [*n* = 133; 11%]; pre = no / post = yes [*n* = 114; 9%]).

### Characteristics

Table 1 details the baseline subject characteristics of the overall cohort and by CTO status. Overall, the mean age of individuals with schizophrenia was 38 (SD 14) years, there were twice as many males (64.3%) as females (35.7%), 89.0% lived in urban areas, the overall mean CCI score was 0.6 (SD 1.3), and 13.3% initiated a SGA-LAI while hospitalized. Compared with the other CTO cohorts, the pre = no / post = yes cohort had a higher proportion of males (71.9%), were younger in age (34 years [SD 14]), with almost one third of individuals between 18 and 24 years of age (32.5%), and had the highest proportion who initiated a SGA-LAI while hospitalized (27.2%). On the index date, the majority of individuals initiated paliperidone (67.6%—71.4%), followed by aripiprazole (16.7%—21.1%), followed by risperidone (11.3%—13.4%).

**Table 1:** Subject characteristics presented for the overall cohort and by CTO status

	Overall cohort (*n* = 1211; 100%)	CTO status			
		pre = no/post = no (*n* = 689; 57%)	pre = yes/post = yes (*n* = 275; 23%)	pre = yes/post = no (*n* = 133; 11%)	pre = no/post = yes (*n* = 114; 9%)
Age, years					
Mean (SD)	38 (14)	39 (14)	38 (14)	38 (12)	34 (14)
Categories, n (%)					
18–24	239 (19.7%)	134 (19.5%)	44 (16.0%)	24 (18.1%)	37 (32.5%)
25–29	196 (16.2%)	101 (14.7%)	58 (21.1%)	18 (13.5%)	19 (16.7%)
30–34	150 (12.4%)	95 (13.8%)	27 (9.8%)	14 (10.5%)	14 (12.3%)
35–39	132 (10.9%)	64 (9.3%)	43 (15.6%)	15 (11.3%)	10 (8.8%)
40–44	124 (10.2%)	64 (9.3%)	26 (9.5%)	24 (18.1%)	10 (8.8%)
45–49	105 (8.7%)	66 (9.6%)	17 (6.2%)	15 (11.3%)	< 10 (< 8.8%)
50–54	101 (8.3%)	63 (9.1%)	22 (8.0%)	12 (9.0%)	< 10 (< 8.8%)
55–59	69 (5.7%)	43 (6.2%)	15 (5.5%)	< 10 (< 7.5%)	< 10 (< 8.8%)
60–64	40 (3.3%)	25 (3.6%)	< 15 (< 5.5%)	< 10 (< 7.5%)	< 10 (< 8.8%)
≥ 65	55 (4.5%)	34 (4.9%)	< 15 (< 5.5%)	< 10 (< 7.5%)	< 10 (< 8.8%)
Sex, n (%)					
Male	779 (64.3%)	436 (63.3%)	177 (64.4%)	84 (63.2%)	82 (71.9%)
Female	432 (35.7%)	253 (36.7%)	98 (35.6%)	49 (36.8%)	32 (28.1%)
Residence, n (%)					
Urban	1078 (89.0%)	608 (88.2%)	252 (91.6%)	118 (88.7%)	100 (87.7%)
Rural	133 (11.0%)	81 (11.8%)	23 (8.4%)	15 (11.3%)	14 (12.3%)
CCI, mean (SD)	0.6 (1.3)	0.7 (1.4)	0.5 (1.0)	0.6 (1.3)	0.6 (1.0)
Hospitalized on index date, n (%)	161 (13.3%)	87 (12.6%)	36 (13.1%)	< 10 (< 7.5%)	31 (27.2%)
Index SGA-LAI, n (%)					
Paliperidone	833 (68.8%)	471 (68.4%)	186 (67.6%)	95 (71.4%)	81 (71.1%)
Aripiprazole	229 (18.8%)	126 (18.3%)	58 (21.1%)	26 (19.5%)	19 (16.7%)
Risperidone	149 (12.3%)	92 (13.4%)	31 (11.3%)	14 (11.3%)	14 (12.3%)

*Abbreviations*: *CCI* Charlson comorbidity index, *CTO* Community treatment order, *SD* Standard deviation, *SGA-LAI* Second generation antipsychotic long-acting injectable. Subject numbers less than 10 need to be redacted as per data privacy standards, and prevented from being back-calculated

### Antipsychotic medication use and adherence

Table 2 details antipsychotic medication use and overall MPR among all subjects within the overall cohort during the 2-year pre-index and post-index periods; MPR according to formulation (oral or injectable) and generation (first or second) are presented for completeness. In addition to the study-defined eligibility criteria of ≥ 1 SGA-LAI dispensation during the post-index period and none during the pre-index period, SGA oral medications were dispensed to 79.8% of the overall cohort during the pre-index period and 69.5% during the post-index period. Respectively, FGA oral and LAIs were dispensed to 10.7% and 15.9% of the overall cohort during the pre-index period, and 9.9% and 5.5% during the post-index period. The overall mean antipsychotic MPR was 0.39 (95% confidence interval [CI] 0.36, 0.41) greater during the 2-year post-index period (0.84 [SD 0.26]) compared with the 2-year pre-index period (0.45 [SD 0.40]). Among the oral antipsychotics, the MPR of FGA and SGA medications were not significantly different between the pre- and post-index periods. Although the MPR of FGA-LAI was low during the study period (0.07 [SD 0.20] pre-index; 0.02 [SD 0.11] post-index), it was 0.05 (95% CI -0.060, -0.038) less in the post-index period. The MPR of SGA-LAI was 0.64 (SD 0.33) during the post-index period.

**Table 2:** Antipsychotic medication use and mean medication possession ratio of the overall cohort during the pre-index and post-index periods

	Pre-index period (*n* = 1,211; 100%)		Post-index period (*n* = 1,211; 100%)		Mean difference [95% CI]
*Received* ≥ *1 dispensation, n (%)*					
Oral					
First generation	130 (10.7%)		120 (9.9%)		
Second generation	966 (79.8%)		841 (69.5%)		
Long acting-injectable					
First generation	192 (15.9%)		67 (5.5%)		
Second generation	N/A (N/A)		1,211 (100%)		
*Medication possession ratio, mean (SD); mean difference [95%CI]*					
Overall	0.45 (0.40)		0.84 (0.26)		**0.39 [0.36, 0.41]**
Oral					
First generation	0.03 (0.14)		0.03 (0.13)		-0.0046 [-0.014, 0.0043]
Second generation	0.40 (0.39)		0.38 (0.41)		-0.019 [-0.044, 0.0065]
Long acting-injectable					
First generation	0.07 (0.20)		0.02 (0.11)		**-0.05 [-0.060, -0.038]**
Second generation	N/A (N/A)		0.64 (0.33)		N/A

**Bolded** mean difference indicates statistically significant difference (*p* < 0.001) between the 2-year post- and 2-year pre-index using paired t-tests. *Abbreviations*: *CI* Confidence interval, *N/A* Not applicable, *SD* standard deviation

Among the CTO cohorts (Additional file 3), the proportion of individuals who received oral (pre-index: 9.5% to 11.5%; post-index: < 7.5% to 15.8%) and LAI (pre-index: 14.7% to 21.1%; post-index: 4.9% to < 8.8%) formulations of FGAs was relatively low both in the pre- and post-index periods, and the proportion who received oral SGAs was relatively high (pre-index: 76.4% to 81.4%; post-index: 60.2% to 82.5%). The overall mean antipsychotic MPR was significantly greater during the 2-year post-index period compared with the 2-year pre-index period among all subjects within each of the CTO subgroups; adherence ranged between 37% (0.37 [SD 0.36]) and 49% (0.49 [SD 0.41]) in the pre-index period and between 80% (0.80 [SD 0.26]) and 92% (0.92 [SD 0.18]) in the post-index period (Additional file 3). Among the oral antipsychotics, the MPR of FGA and SGA medications were not significantly different between the pre- and post-index periods, with the exception of the pre = yes / post = no CTO cohort that had a lower MPR of oral FGAs in the post-index (1% MPR) period compared with the pre-index (2% MPR) period (0.01 [SD 0.06] versus 0.02 [SD 0.10]; -0.009 [95% CI -0.018, -0.001]), measured among all subjects in the cohort. Regarding FGA-LAIs, MPR was significantly lower in the post-index period than the pre-index period, with the exception of the pre = yes / post = no CTO cohort that displayed no significant pre-post difference.

### Healthcare resource utilization

**Fig. 2:**
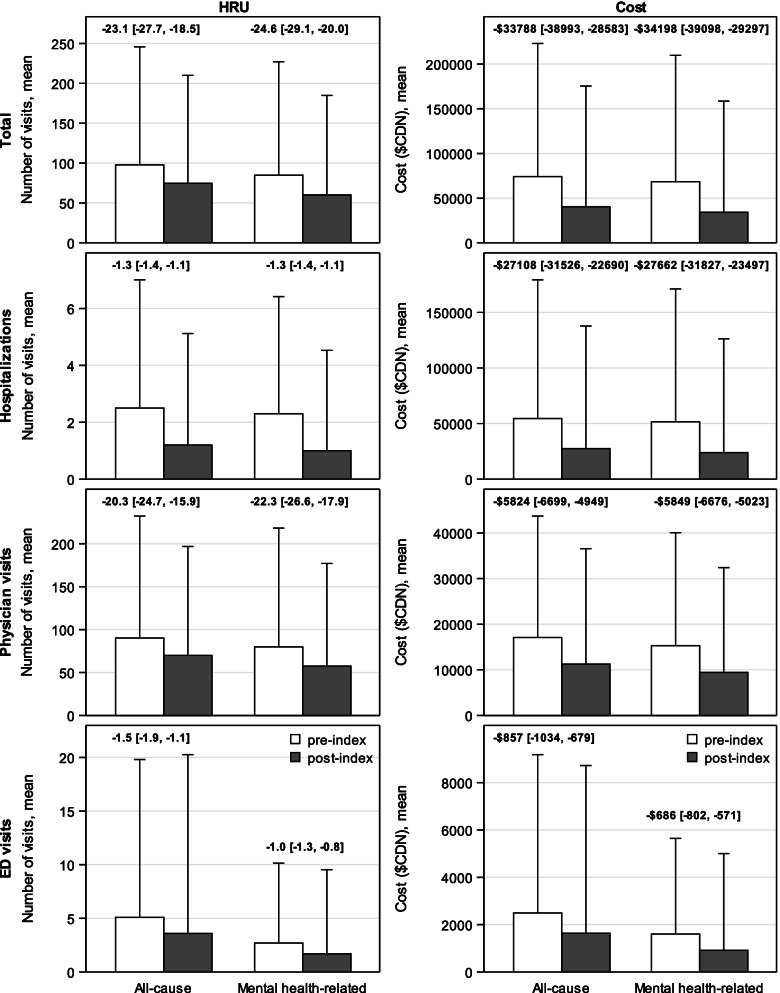
Mean per-patient health care resource utilization and cost among the overall cohort. All comparative differences (mean difference [95% confidence interval]) between the post-index and pre-index periods were statistically significant (*p* < 0.001) using paired t-tests. Abbreviations: CDN = Canadian; ED = emergency department; HRU = healthcare resource utilization

Figure 2 show the mean per patient number and comparative differences of all-cause and mental health-related total visits, as well as hospitalizations, physician visits, and ED visits for the overall cohort between the pre-index and post-index periods. During the study period, all-cause physician visits were most common (97.8 [SD 75.5] pre-index, 74.7 [SD 69.1] post-index), followed by ED visits (5.1 [SD 7.5] pre-index, 3.6 [SD 8.5] post-index), and hospitalizations (2.5 [SD 2.3] pre-index, 1.2 [SD 2.0] post-index) within the overall cohort. The mean number of total all-cause HRU visits was significantly lower by 23.6% (74.7 [SD 69.1] versus 97.8 [SD 75.5] visits; mean difference -23.1 [95% CI -27.7, -18.5]) in the post-index period compared with the pre-index period. The mean difference in specific types of HRU visits between the post-index and the pre-index periods was also significantly lower for hospitalizations (-1.3 [95% CI -1.4, -1.1]), physician visits (-20.3 [95% CI -24.7, -15.9]), and ED visits (-1.5 [95% CI -1.9, -1.1]). For the most part, mental health-related HRU visits within the overall cohort comprised the majority of all-cause total (87% and 81%), hospital (92% and 83%), physician visits (88% and 82%), and ED visits (53% and 47%) during pre-index and post-index periods, respectively. The total mean number of mental health-related visits within the overall cohort was significantly lower in the post-index period compared with the pre-index period (84.8 [SD 72.6] versus 60.2 [SD 63.6]; mean difference -24.6 [95%CI -29.1, -20.0]), as well as hospitalizations, physician visits, and ED visits (*p* < 0.001).

Among the CTO cohorts, all-cause and mental health-related total, hospital, physician, and ED visits were significantly lower in the post-index period compared with the pre-index period, with the exception of the pre = no / post = yes CTO cohort that displayed no significant differences in HRU visits between the pre- and post-index periods (Additional file 4). The pre = yes / post = no CTO subgroup consistently showed the largest difference in visits across all-cause and mental health-related total HRU, as well as hospitalizations, physician visits, and ED visits (Additional file 4).

### Health resource utilization costs

Figure 2 also shows the mean cost ($CDN) and comparative differences of all-cause and mental health-related total HRU costs, as well as hospitalization, physician visit, and ED visit costs for the overall cohort between the pre-index and post-index periods. All-cause hospitalizations incurred the highest costs ($54,560 [SD $63,681] pre-index, $27,453 [SD $56,298] post-index), followed by physician visits costs ($17,077 [SD $13,582] pre-index, $11,253 [SD $12,911] post-index), and ED costs ($2,494 [SD $3,413] pre-index, $1,638 [SD $3,615] post-index) during the study period. The mean cost difference between the post-index period and the pre-index period was significantly lower for all-cause total HRU (-$33,788 [95% -$38,993, -$28,583]), and the specific costs for hospitalizations (-$27,108 [95% CI -$31,526, -$22,690]), physician visits (-$5,824 [95% CI -$6,699, -$4,949]), and ED visits (-$857 [95% CI -$1,034, -$679]) within the overall cohort. Mental health-related HRU costs within the overall cohort comprised the majority of all-cause total (92% and 85%), hospital (94% and 87%), physician visit (89% and 84%), and ED visit (64% and 56%) costs during pre-index and post-index periods, respectively. The total cost of mental health-related visits within the overall cohort was significantly lower in the post-index period compared with the pre-index period ($34,205 [SD $63,428] CDN versus $68,403 [SD $72,088] CDN; mean difference -$34,198 [95%CI -$39,098, -$29,297]), as well as hospitalizations, physician visits, and ED visits (*p* < 0.001). Statistical results were the same when median costs were compared using the Wilcoxon signed-rank test (Additional file 5).

Among the CTO cohorts, all-cause and mental health-related total, hospital, physician, and ED visit costs were significantly lower in the post-index period compared with the pre-index period (*p* < 0.001), with the exception of the pre = no / post = yes CTO cohort that displayed no significant differences in HRU costs (Table 3). The pre = yes / post = no CTO subgroup consistently showed the largest difference in cost across all-cause and mental health-related total HRU, as well as hospitalizations, physician visits, and ED visits (Table 3). Statistical results were the same when median costs were compared using the Wilcoxon signed-rank test (Additional file 5).

**Table 3:** Mean healthcare costs during the pre- and post-index periods among the CTO cohorts

	CTO status							
	pre = no / post = no (*n* = 689; 57%)		pre = yes / post = yes (*n* = 275; 23%)		pre = yes / post = no (*n* = 133; 11%)		pre = no / post = yes (*n* = 114; 9%)	
	pre-index	post-index	pre-index	post-index	pre-index	post-index	pre-index	post-index
All-Cause, $CDN								
Total, mean (standard deviation)	$61,281 (72,902)	$34,387 (65,056)	$91,342 (74,924)	$51,324 (80,380)	$100,699 (82,557)	$15,987 (22,746)	$79,285 (72,358)	$78,268 (78,204)
difference [95%CI]	**-$26,894 [-33472, -20316]**		**-$40,018 [-51363, -28673]**		**-$84,712 [-98938, -70486]**		-$1017 [-19082, 17048]	
Hospitalizations, mean (standard deviation)	$44,871 (62,059)	$23,016 (52,911)	$66,326 (60,263)	$36,392 (67,225)	$76,597 (72,378)	$7913 (17,777)	$59,026 (59,563)	$55,499 (63,312)
difference [95%CI]	**-$21,855 [-27526, -16185]**		**-$29,934 [-39438, -20430]**		**-$68,684 [-81158, -56210]**		-$3527 [-18210, 11156]	
Physician visits, mean (standard deviation)	$14,013 (12,111)	$9759 (12,641)	$22,347 (15,303)	$13,266 (13,551)	$21,861 (12,627)	$7300 (5654)	$17,297 (13,275)	$20,039 (14,477)
difference [95%CI]	**-$4254 [-5286, -3222]**		**-$9081 [-11093, -7069]**		**-$14,561 [-16684, -12439]**		$2742 [-682, 6166]	
ED visits, mean (standard deviation)	$2396 (3768)	$1612 (4258)	$2669 (2892)	$1666 (2400)	$2241 (1988)	$774 (1412)	$2962 (3593)	$2730 (3335)
difference [95%CI]	**-$785 [-1038, -531]**		**-$1002 [-1310, -695]**		**-$1467 [-1811, -1123]**		-$232 [-933, 469]	
Mental health-related, $CDN								
Total, mean (standard deviation)	$55,360 (68,443)	$27,965 (59,719)	$86,532 (73,380)	$44,222 (74,477)	$92,753 (73,726)	$13,974 (22,046)	$75,092 (71,688)	$71,361 (70,648)
difference [95%CI]	**-$27,395 [-33667, -21122]**		**-$42,311 [-53047, -31575]**		**-$78,779 [-91476, -66082]**		-$3731 [-20470, 13008]	
Hospitalizations, mean (standard deviation)	$41,827 (59,543)	$19,300 (49,347)	$64,106 (59,601)	$31,838 (61,986)	$70,814 (63,850)	$7188 (17,376)	$57,283 (59,364)	$51,652 (57,815)
difference [95%CI]	**-$22,527 [-27964, -17090]**		**-$32,268 [-41216, -23319]**		**-$63,626 [-74636, -52616]**		-$5631 [-19328, 8067]	
Physician visits, mean (standard deviation)	$12,128 (10,663)	$78,456 (11,155)	$20,546 (14,677)	$11,340 (12,711)	$20,243 (12,104)	$6316 (5398)	$15,773 (12,989)	$17,972 (13,319)
difference [95%CI]	**-$4282 [-5248, -3316]**		**-$9206 [-11138, -7274]**		**-$13,927 [-15957, -11897]**		$2200 [-996, 5395]	
ED visits, mean (standard deviation)	$1405 (2080)	$819 (2314)	$1881 (1978)	$1044 (1769)	$1696 (1417)	$470 (967)	$2036 (2588)	$1736 (2066)
difference [95%CI]	**-$586 [-742, -430]**		**-$837 [-1055, -619]**		**-$1226 [-1472, -980]**		-$300 [-799, 198]	

**Bolded** mean difference indicates statistically significant difference (*p* < 0.001) between the 2-year post- and 2-year pre-index periods using paired t-tests. *Abbreviations*: *CDN* Canadian, *CI* Confidence interval, *CTO* Community treatment order, *ED* Emergency department

## Discussion

In this retrospective, non-interventional, single arm study of 1,211 adults with schizophrenia, real-world antipsychotic medication adherence, and HRU and associated costs were compared between the 2-year period before and after SGA-LAI initiation using administrative data from April 1, 2012 to March 31, 2018 in Alberta, Canada; subgroup analysis was performed based on CTO status. Results showed that among adults with schizophrenia, overall adherence to antipsychotic medication was greater, and total HRU and associated costs, including all-cause and mental health-related hospitalizations, physician visits, and ED visits were lower during the 2-year period following initiation of a SGA-LAI compared to the 2-year period before. Regarding CTO status, 80% of individuals persisted with the same CTO status throughout the pre- and post-index periods (pre = no / post = no; pre = yes / post = yes), and had similar decreases in HRU and costs as the overall cohort. Among those with a change in CTO status during the study period, the pre = yes / post = no CTO cohort consistently showed the largest differences in HRU and costs between the pre- (higher) and post- (lower) index periods; the pre = no / post = yes CTO cohort displayed no significant differences in HRU visits and costs between the pre- and post-index periods. Collectively, results of this study indicate that SGA-LAIs may be an effective strategy for assisting with long-term antipsychotic medication adherence, and while causality cannot be established, lower HRU and associated costs to the healthcare system occurred over a 2-year period after SGA-LAI initiation; however, these can vary depending on CTO status.

Approaches to improve treatment adherence among individuals living with schizophrenia include LAI formulations of antipsychotics [10, 11]. In support of this treatment approach, we found higher overall antipsychotic medication adherence after SGA-LAI initiation compared with before. Although not the focus of this study, we also found that the proportion of individuals who had used an oral SGA was quite high in the pre- and post-index period, while those who used a FGA both oral or LAI was low. These findings may reflect prescribing practices where individuals are generally initiated on a LAI formulation of the corresponding current oral antipsychotic, and while polypharmacy between different types and formulations of antipsychotics is not uncommon, the same type of LAI and oral are generally prescribed together [24–26]. These findings may warrant inquiry in future work examining antipsychotic medication treatment patterns and prescribing practices among individuals initiating LAIs; for those also under an active CTO, understanding which antipsychotics, if any, are included within the treatment plan would provide additional insight.

Results from the overall study cohort are consistent with recent observational studies, which have reported lower HRU and costs after initiation of LAI antipsychotic medication compared with before among individuals with schizophrenia [27, 28]. Among individuals who initiated SGA-LAI antipsychotic medication in Quebec, Canada, Stip et al*.* (2018) found that the number of hospitalizations and ED visits were lower in the 1-year period after initiation compared to the year prior, and that the observed 64% reduction in total HRU costs was primarily driven by the pre-post difference in hospitalization costs [27], which has been reported to represent the largest proportion of direct healthcare costs for schizophrenia [3]. However, in contrast to Stip et al*.* (2018) who also reported no significant pre-post differences in physician visits, we found that the number of physician visits were significantly lower in the post- versus pre-index period, which may be partially due to the longer observation period employed in the current study, as a 2-year observation period has been suggested to result in a higher likelihood of detecting a difference compared with 1-year periods [15]. Collectively, our results support and extend previous findings by showing that HRU and associated costs, made up primarily of hospitalization costs, were lower during the 2-year period after SGA-LAI initiation compared with before, which is a longer observation period than most previous reports [15].

Another unique aspect of this study was that analysis was also performed based on CTO status during the study period, of which 43% of individuals received at least one active CTO. The intention of a CTO is to assist with improving treatment adherence in patients with greater illness severity. To this end, O’Brien et al*.* (2009) found that in Ontario, Canada, patients who were placed on a CTO had severe and persistent mental illness [29]. Not surprisingly, varying patterns of HRU and associated costs were found among the different CTO cohorts. While 80% of individuals persisted with the same CTO status throughout the pre- and post-index periods (57% pre = no / post = no; 23% pre = yes / post = yes) and had similar decreases in HRU and costs as the overall cohort, 20% of individuals had a CTO status change during the study period (11% pre = yes / post = no; 9% pre = no / post = yes) along with associated HRU and costs that varied from the overall cohort. The pre = yes / post = no CTO cohort displayed the largest mean difference in all measured HRU and associated costs between the pre-index period, which was higher, and the post-index period, which was lower. Considering that these individuals had an overall antipsychotic medication adherence of 92% in the post-index period (compared with 40% in the pre-index period), it is possible that after SGA-LAI initiation a stable phase of illness may have been attained where symptoms and functioning significantly improved [6]; however this cannot be confirmed without clinical evaluative data. The pre = no / post = yes CTO cohort displayed HRU and costs that were distinctive from the overall cohort, as well as the other CTO cohorts. Although overall antipsychotic medication adherence was greater during the 2-year post-index period (83%) than beforehand (46%) in this cohort, no significant pre-post differences in HRU and associated costs were observed. Vincent et al*.* (2017) conducted a mirror-image study investigating individuals living with schizophrenia or schizoaffective disorder who initiated a SGA-LAI while hospitalized in Quebec, Canada [14]. The authors found that initiating a SGA-LAI improved adherence, but those with a pending CTO had a lengthy index hospital stay while awaiting CTO activation that may have contributed to the pre-post cost-neutral results. Considering that the pre = no / post = yes CTO cohort had the highest proportion of individuals who were hospitalized when a SGA-LAI was initiated, perhaps this may have contributed to the finding of no significant pre-post differences in HRU and associated costs within this CTO cohort; the smaller sample size of this cohort may have also reduced power to detect true differences.

This study has several important strengths, including the large size, population-based design, incorporation of CTO status, and long observation periods. However, this study is also subject to a number of limitations that should be taken into consideration when interpreting results. 1) While individuals with schizophrenia were identified using a validated case definition, administrative health data was used as opposed to medical records, and therefore there is a potential for misclassification of the study groups or measures. 2) Pre-post study designs lack a control group, hence the outcome patterns in the counter-factual patients who are similar to our patient group in all aspects except SGA-LAI initiation are unknown; this is particularly important when interpreting outcomes in patients who had a change in CTO status, which may be an indicator of other factors including disease instability. 3) PIN data only captures dispensations of prescription medications, and therefore actual medication up-take by patients is unknown. However, since LAI antipsychotics are injected by healthcare professionals, the dispensation of SGA-LAIs in this study should be highly representative of the actual uptake. 4) Use of over-the-counter medications, prescription medications provided in a hospital or secondary care setting, and other non-pharmacotherapy treatments are not captured within provincial administrative data and therefore, not reported.

## Conclusions

Among adults with schizophrenia, antipsychotic medication adherence was higher, and HRU and associated costs primarily comprised of hospitalization costs, were lower during the 2-year period after SGA-LAI initiation as compared to before, extending findings of previous mirror-image and naturalistic studies. CTOs should be considered in research studies involving individuals with schizophrenia as they are commonly used in this population and our results show that HRU and associated costs can vary depending on CTO status.

## Supplementary Information

**Additional file 1. **Diagnostic codes used within the schizophrenia case finding algorithm.

**Additional file 2. **Identification of oral and long-acting injectable antipsychotics based on anatomical therapeutic chemical classification and/or drug identification number.

**Additional file 3. **Antipsychotic medication use and mean possession ratio among the CTO cohorts.

**Additional file 4. **Healthcare resource utilization during the pre- and post-index periods among the CTO cohorts.

**Additional file 5. **Median healthcare costs during the pre- and post-index periods among the overall and CTO cohorts.

## Data Availability

The data that support the findings of this study are not available. The data custodians, Alberta Health Services and Alberta Health do not allow users of the data to publish the data. Please contact the corresponding author for requests related to the data used in this study.

## Abbreviations

AHCIP: Alberta Health Care Insurance Plan
CCI: Charlson Comorbidity Index
CDN: Canadian
CI: Confidence interval
CSHS: Cost of a standard hospital stay
CTO: Community treatment order
DAD: Discharge Abstract Database
ED: Emergency department
FGA: First-generation antipsychotic
HRU: Healthcare resource utilization
ICD-9-CM: International Classification of Disease—Version 9—Canadian Modification
ICD-10-CA: International Classification of Disease—Version 10—Canadian Enhancement
IQR: Interquartile range
LAI: Long-acting injectable
MPR: Medication possession ratio
N/A: Not applicable
NACRS: National Ambulatory Care Reporting System
PIN: Pharmaceutical Information Network
RIW: Resource intensity weight
SD: Standard deviation
SGA: Second-generation antipsychotic
