# Supplementary material for: Health resource utilization and cost before versus after initiation of second-generation long-acting injectable antipsychotics among adults with schizophrenia in Alberta, Canada: a retrospective, observational single-arm study
Source: BMC Psychiatry. 2022 Jul 2;22:444. doi: 10.1186/s12888-022-04075-y (PMC9250716; doi:10.1186/s12888-022-04075-y)
Supplement: Supplementary file 1 — Additional file 1. Diagnostic codes used within the schizophrenia case finding algorithm. [file 12888_2022_4075_MOESM1_ESM.docx]

Additional file 1. Diagnostic codes used within the schizophrenia case finding algorithm.

|  | ICD-9-CM Codes | ICD-10-CA Codes |
| --- | --- | --- |
| Schizophrenia | 295.0, 295.1, 295.2, 295.3, 295.4, 295.6, 295.8, 295.9 | F20 |
| Schizotypal disorder | 301.2 | F21 |
| Schizoaffective disorder | 295.7 | F25 |
| Psychotic disorder not elsewhere classified | 298.8 | F29 |

Abbreviations: ICD-9-CM = International Classification of Disease - Version 9 - Canadian Modification; ICD-10-CA = International Classification of Disease - Version 10 - Canadian Enhancement.
