# Supplementary material for: Health resource utilization and cost before versus after initiation of second-generation long-acting injectable antipsychotics among adults with schizophrenia in Alberta, Canada: a retrospective, observational single-arm study
Source: BMC Psychiatry. 2022 Jul 2;22:444. doi: 10.1186/s12888-022-04075-y (PMC9250716; doi:10.1186/s12888-022-04075-y)
Supplement: Supplementary file 2 — Additional file 2. Identification of oral and long-acting injectable antipsychotics based on anatomical therapeutic chemical classification and/or drug identification number. [file 12888_2022_4075_MOESM2_ESM.docx]

Additional file 2. Identification of oral and long-acting injectable antipsychotics based on anatomical therapeutic chemical classification and/or drug identification number.

|  | Oral  ATC code | Long-acting injectable  DIN code |
| --- | --- | --- |
| First generation | | |
| Chlorpromazine  Fluphenazine  Flupentixol  Haloperidol  Loxapine  Methotrimeprazine  Periciazine  Perphenazine  Pimozide  Pipotiazine  Prochlorperazine  Thioproperazine  Thiothixene  Trifluoperazine  Zuclopenthixol | N05AA01  N05AB02  N05AF01  N05AD01  N05AH01  N05AA02  N05AC01  N05AB03  N05AG02  N05AB04  N05AB08  N05AF04  N05AB06  N05AF05 | 00755575  02156032, 02156040  02130300  01926667, 01926675  02230405, 02230406 |
|  |  |  |
| Second generation | | |
| Aripiprazole  Asenapine  Brexpiprazole  Clozapine  Lurasidone  Olanzapine  Paliperidone  Quetiapine  Risperidone  Ziprasidone | N05AX12  N05AH05  N05AX16  N05AH02  N05AE05  N05AH03  N05AX13  N05AH04  N05AX08  N05AE04 | 02420864, 02420872  02354217, 02354225, 02354233, 02354241, 02455943, 02455986, 02455994, 02456001  02298465, 02255707, 02255723, 02255758 |

An ATC code contains all DINs for a specific drug, and therefore the oral antipsychotic drugs were identified by an ATC code with the long-acting injectable DIN codes excluded. Abbreviations: ATC = anatomical therapeutic chemical; DIN = drug identification number.
