# Supplementary material for: Health resource utilization and cost before versus after initiation of second-generation long-acting injectable antipsychotics among adults with schizophrenia in Alberta, Canada: a retrospective, observational single-arm study
Source: BMC Psychiatry. 2022 Jul 2;22:444. doi: 10.1186/s12888-022-04075-y (PMC9250716; doi:10.1186/s12888-022-04075-y)
Supplement: Supplementary file 3 — Additional file 3. Antipsychotic medication use and mean possession ratio among the CTO cohorts. [file 12888_2022_4075_MOESM3_ESM.docx]

Additional file 3. Antipsychotic medication use and mean possession ratio among the CTO cohorts.

|  | CTO status | | | | | | | | |
| --- | --- | --- | --- | --- | --- | --- | --- | --- | --- |
|  | pre=no / post=no  (n=689; 57%) | | pre=yes / post=yes  (n=275; 23%) | | pre=yes / post=no  (n=133; 11%) | | pre=no / post=yes  (n=114; 9%) | | |
|  | pre-index | post-index | pre-index | post-index | pre-index | post-index | pre-index | post-index | |
| *Received ≥1 dispensation, n (%)* | | | | |  |  |  |  | |
| Oral |  |  |  |  |  |  |  |  | |
| First generation | 81 (11.8%) | 72 (10.4%) | 25 (9.1%) | 27 (9.8%) | 12 (9.0%) | <10 (<7.5%) | 12 (10.5%) | 18 (15.8%) | |
| Second generation | 561 (81.4%) | 489 (71.0%) | 210 (76.4%) | 178 (64.7%) | 105 (78.9%) | 80 (60.2%) | 90 (78.9%) | 94 (82.5%) | |
| Long acting-injectable | | |  |  |  |  |  |  | |
| First generation | 101 (14.7%) | 34 (4.9%) | 44 (16.0%) | 17 (6.2%) | 23 (17.3%) | <10 (<7.5%) | 24 (21.1%) | <10 (<8.8%) | |
| Second generation | N/A (N/A) | 689 (100%) | N/A (N/A) | 275 (100%) | N/A (N/A) | 133 (100%) | N/A (N/A) | 114 (100%) | |
| *Medication possession ratio, mean (SD); mean difference [95% CI]* | | | | | | | | | |
| Overall | 0.49 (0.41) | 0.84 (0.27) | 0.37 (0.36) | 0.80 (0.26) | 0.40 (0.38) | 0.92 (0.18) | 0.46 (0.41) | 0.83 (0.23) | |
|  | **0.34 [0.31, 0,38]** | | **0.44 [0.39, 0.48]** | | **0.52 [0.44, 0.59]** | | **0.37 [0.29, 0.44]** | | |
| Oral |  |  |  |  |  |  |  |  | |
| First generation | 0.04 (0.16) | 0.03 (0.15) | 0.02 (0.11) | 0.02 (0.11) | 0.02 (0.10) | 0.01 (0.06) | 0.04 (0.14) | 0.03 (0.10) | |
|  | -0.005 [-0.018, -0.008] | | 0.002 [-0.016, 0.020] | | **-0.009 [-0.018, -0.001]** | | -0.011 [-0.037, 0.0.15] | | |
| Second generation | 0.44 (0.40) | 0.41 (0.42) | 0.32 (0.34) | 0.31 (0.38) | 0.37 (0.39) | 0.36 (0.43) | 0.38 (0.40) | 0.40 (0.38) | |
|  | -0.030 [-0.065, -0.004] | | -0.005 [-0.052, 0.042] | | -0.016 [-0.100, 0.068] | | 0.017 [-0.056, 0.091] | | |
| Long acting-injectable | | |  |  |  |  |  |  | |
| First generation | 0.07 (0.21) | 0.02 (0.10) | 0.06 (0.17) | 0.02 (0.10) | 0.04 (0.15) | 0.02 (0.13) | 0.11 (0.26) | 0.02 (0.09) | |
|  | **-0.05 [-0.07, -0.04]** | | **-0.03 [-0.05, -0.02]** | | -0.02 [-0.05, 0.01] | | **-0.09 [-0.13, -0.04]** | | |
| Second generation | N/A (N/A) | 0.63 (0.34) | N/A (N/A) | 0.61 (0.32) | N/A (N/A) | 0.79 (0.28) | N/A (N/A) | |  |
|  | N/A (N/A) | | N/A (N/A) | | N/A (N/A) | | N/A (N/A) | | |

**Bolded** mean difference indicates statistically significant difference (p<0.001) between the 2-year post- and 2-year pre-index periods using paired t-tests. Abbreviations: CI = confidence interval; CTO = community treatment order; SD = standard deviation.
