# Supplementary material for: Health resource utilization and cost before versus after initiation of second-generation long-acting injectable antipsychotics among adults with schizophrenia in Alberta, Canada: a retrospective, observational single-arm study
Source: BMC Psychiatry. 2022 Jul 2;22:444. doi: 10.1186/s12888-022-04075-y (PMC9250716; doi:10.1186/s12888-022-04075-y)
Supplement: Supplementary file 4 — Additional file 4. Healthcare resource utilization during the pre- and post-index periods among the CTO cohorts. [file 12888_2022_4075_MOESM4_ESM.docx]

Additional file 4. Healthcare resource utilization during the pre- and post-index periods among the CTO cohorts.

|  | CTO status | | | | | | | | | | | | |  |  |
| --- | --- | --- | --- | --- | --- | --- | --- | --- | --- | --- | --- | --- | --- | --- | --- |
|  | pre=no / post=no  (n=689; 57%) | | | | pre=yes / post=yes  (n=275; 23%) | | | | pre=yes / post=no  (n=133; 11%) | | | pre=no / post=yes  (n=114; 9%) | |  |  |
|  | pre-index | | post-index | | pre-index | | post-index | | pre-index | | post-index | pre-index | post-index |  |  |
| Mean (standard deviation); difference [95% confidence interval] | | | | | | | |  | |  | |  |  | |  |
| All-Cause |  |  | |  | |  | |  | |  | |  |  | |  |
| Total | 86.7  (73.8) | | 69.0  (69.5) | | 116.4  (77.5) | | 84.4  (73.4) | | 117.8  (69.3) | | 53.7  (35.3) | 96.2  (75.2) | 110.3  (70.7) | |  |
|  | **-17.8 [-23.5, -12.0]** | | | | **-32.0 [-42.3, -21.7]** | | | | **-64.1 [-75.6, -52.6]** | | | 14.1 [-2.9, 31.1] | | |  |
| Hospitalizations | 2.1  (2.2) | | 1.0  (1.8) | | 3.0  (2.3) | | 1.5  (2.3) | | 2.9  (1.9) | | 0.5  (0.9) | 3.0  (2.6) | 2.6  (2.4) | |  |
|  | **-1.1 [-1.3, -0.9]** | | | | **-1.5 [-1.8, -1.2]** | | | | **-2.4 [-2.7, -2.1]** | | | -0.5 [-1.0, 0.1] | | |  |
| Physician visit | 79.6  (70.3) | | 64.3  (64.8) | | 108.1  (74.3) | | 79.4  (69.4) | | 110.6  (67.8) | | 51.3  (33.8) | 87.0  (73.2) | 102.2  (66.7) | |  |
|  | **-15.3 [-20.8, -9.9]** | | | | **-28.6 [-38.6, -18.7]** | | | | **-59.3 [-70.6, -47.9]** | | | 15.1 [-1.2, 31.4] | | |  |
| ED visits | 5.0  (8.1) | | 3.6  (10.1) | | 5.4  (7.0) | | 3.5  (5.5) | | 4.3  (4.5) | | 1.9  (3.8) | 6.1  (7.8) | 5.6  (7.3) | |  |
|  | **-1.4 [-2.0, -0.8]** | | | | **-1.8 [-2.5, -1.1]** | | | | **-2.4 [-3.2, -1.7]** | | | -0.6 [-2.1, 1.0] | | |  |
|  |  |  | |  | |  | |  | |  | |  |  | |  |
| Mental health-related | |  | |  | |  | |  | |  | |  |  | |  |
| Total | 72.5  (69.0) | | 53.3  (62.7) | | 104.6  (76.1) | | 70.1  (69.0) | | 107.9  (67.8) | | 44.1  (32.9) | 84.5  (74.4) | 96.8  (66.4) | |  |
|  | **-19.2 [-24.7, -13.6]** | | | | **-34.4 [-44.6, -24.3]** | | | | **-63.8 [-75.4, -52.2]** | | | 12.4 [-4.2, 28.9] | | |  |
| Hospitalizations | 1.9  (1.9) | | 0.8  (1.6) | | 2.8  (2.3) | | 1.3  (2.2) | | 2.7  (1.8) | | 0.4  (0.8) | 2.8  (2.3) | 2.3  (2.1) | |  |
|  | **-1.1 [-1.2, -0.9]** | | | | **-1.5 [-1.8, -1.2]** | | | | **-2.3 [-2.6, -2.0]** | | | -0.5 [-1.0, 0.0] | | |  |
| Physician visit | 68.2  (67.2) | | 51.0  (60.4) | | 98.6  (73.7) | | 67.0  (65.7) | | 102.4  (66.6) | | 42.8  (32.0) | 78.2  (73.4) | 91.5  (64.1) | |  |
|  | **-17.2 [-22.6, -11.8]** | | | | **-31.6 [-41.5, -21.7]** | | | | **-59.6 [-71.0, -48.2]** | | | 13.3 [-2.8, 29.3] | | |  |
| ED visits | 2.4  (3.9) | | 1.5  (4.5) | | 3.2  (3.8) | | 1.9  (3.4) | | 2.8  (2.4) | | 0.9  (1.9) | 3.5  (4.7) | 3.0  (3.7) | |  |
|  | **-0.9 [-1.2, -0.6]** | | | | **-1.3 [-1.7, -0.9]** | | | | **-1.9 [-2.3, -1.4]** | | | -0.4 [-1.3, 0.5] | | |  |

**Bolded** mean difference indicates statistically significant difference (p<0.001) between the 2-year post- and 2-year pre-index periods using paired t-tests. Abbreviations: CTO = community treatment order; ED = emergency department.
