# Supplementary material for: Health resource utilization and cost before versus after initiation of second-generation long-acting injectable antipsychotics among adults with schizophrenia in Alberta, Canada: a retrospective, observational single-arm study
Source: BMC Psychiatry. 2022 Jul 2;22:444. doi: 10.1186/s12888-022-04075-y (PMC9250716; doi:10.1186/s12888-022-04075-y)
Supplement: Supplementary file 5 — Additional file 5. Median healthcare costs during the pre- and post-index periods among the overall and CTO cohorts. [file 12888_2022_4075_MOESM5_ESM.docx]

Additional file 5. Median healthcare costs during the pre- and post-index periods among the overall and CTO cohorts.

|  | Overall cohort  (n=1211; 100%) | | CTO status | | | | | | | |
| --- | --- | --- | --- | --- | --- | --- | --- | --- | --- | --- |
|  |  |  | pre=no / post=no  (n=689; 57%) | | pre=yes / post=yes  (n=275; 23%) | | pre=yes / post=no  (n=133; 11%) | | pre=no / post=yes  (n=114; 9%) | |
|  | pre-index | post-index | pre-index | post-index | pre-index | post-index | pre-index | post-index | pre-index | post-index |
| All-Cause, $CDN |  |  |  |  |  |  |  |  |  |  |
| Total, median  (IQR) | **$ 51742 (26540-95372)** | **$11001  (3933-45409)** | **$39437 (20570-74190)** | **$7742 (3352-34528)** | **$68779 (39237-114184)** | **$16671  (4628-66384)** | **$81411  (45793-128755)** | **$6407  (3970-21691)** | $59589 (37200-101433) | $55909  (28962-103949) |
|  |  | |  | |  | |  | |  | |
| Hospitalizations, median  (IQR) | **$33219**  **(16609- 71421)** | **$0**  **(0-28908)** | **$25481  (10605-53469)** | **$0  (0-19206)** | **$47598 (26837-83047)** | **$5058 (0-46048)** | **$59747 (32292-96392)** | **$0 (0-11087)** | $42985 (21867-79730) | $34613 (16609-66437) |
|  |  | |  | |  | |  | |  | |
| Physician visits, median  (IQR) | **$13760 (7801-22334)** | **$6749  (3461-13994)** | **$11065  (6055-18041)** | **$5439 (3091-11684)** | **$18530 (12246-28224)** | **$8183 (4232-17266)** | **$18511**  **(12148-29280)** | **$5722**  **(3710-9237)** | $13971  (7919-22775) | $15706  (10431-26558) |
|  |  | |  | |  | |  | |  | |
| ED visits, median  (IQR) | **$1527**  **(664-2940)** | **$664 (0-1964)** | **$1327 (664-2744)** | **$612 (0-1802)** | **$1844**  **(949-3318)** | **$664**  **(0-2294)** | **$1511**  **(823-2856)** | **$174**  **(0-949)** | $1930  (983-3578) | $1613  (664-3171) |
| Mental health-related, $CDN | |  |  |  |  |  |  |  |  |  |
| Total, median  (IQR) | **$47563**  **(23779-87752)** | **$6406**  **(2745-36292)** | **$34629**  **(18055-69295)** | **$4409**  **(2163-26731)** | **$64963**  **(37559-104014)** | **$9162**  **(3370-53505)** | **$77736**  **(44331-123609)** | **$5268**  **(3183-18165)** | $55135  (27984-99299) | $48790  (26908-88685) |
|  |  | |  | |  | |  | |  | |
| Hospitalizations, median  (IQR) | **$33219**  **(16609-66002)** | **$0**  **(0-23930)** | **$22893**  **(10605-49828)** | **$0**  **(0-16609)** | **$44730**  **(22896-78476)** | **$0**  **(0-38276)** | **$55244**  **(30989-93963)** | **$0**  **(0-6571)** | $40869  (16609-79322) | $33219  (16609-65251) |
|  |  | |  | |  | |  | |  | |
| Physician visits, median  (IQR) | **$12042**  **(6584-19835)** | **$5225**  **(2546-11273)** | **$9588**  **(4648-16120)** | **$4021**  **(2002-8840)** | **$16814**  **(11298- 25569)** | **$6142**  **(3258-15105)** | **$17424**  **(11881-27329)** | **$5080**  **(3052-7244)** | $12671  (7209-20951) | $13764  (9596-23346) |
|  |  | |  | |  | |  | |  | |
| ED visits, median  (IQR) | **$1138**  **(664-1991)** | **$0**  **(0-1138)** | **$687**  **(475-1613)** | **$0**  **(0-897)** | **$1327**  **(664-2454)** | **$475**  **(0-1327)** | **$1327**  **(663-2265)** | **$0**  **(0-664)** | $1327  (664-2276) | $1138  (664-2276) |
|  |  | |  | |  | |  | |  | |

**Bolded** difference indicates statistically significant difference (p<0.001) between the 2-year post- and 2-year pre-index periods using Wilcoxon matched-pair sign rank tests. Abbreviations: CDN = Canadian; CTO = community treatment order; ED = emergency department; IQR = interquartile range.
